# Primary leiomyoma in the hepatorenal space: a case report

**DOI:** 10.1093/jscr/rjaf062

**Published:** 2025-02-16

**Authors:** Haoyang Huang, Dingwei Xu, Jie Huang

**Affiliations:** Department of Hepatobiliary and Pancreatic Surgery, The Second Affiliated Hospital of Kunming Medical University, Kunming 650101, China; Department of Hepatobiliary and Pancreatic Surgery, The Second Affiliated Hospital of Kunming Medical University, Kunming 650101, China; Department of Hepatobiliary and Pancreatic Surgery, The Second Affiliated Hospital of Kunming Medical University, Kunming 650101, China

**Keywords:** misdiagnosed abdominal tumors, surgical technique, case report

## Abstract

Leiomyomas can occur in various organs and tissues throughout the body, but reports of their occurrence in the hepatorenal space are rare. The video presents a comprehensive breakdown of the diagnostic procedures and surgical resection procedures. Emphasizing the key strategies for easy misdiagnosis. For leiomyomas occurring in rare sites, the compression on surrounding organs often leads to blurred boundaries in imaging, making it difficult to determine their origin and resulting in misdiagnosis. For rare tumors located at the junction areas, in order to achieve better treatment outcomes, abdominal exploration and intraoperative multidisciplinary consultations should be carried out under sufficient preoperative preparations.

## Introduction

Leiomyoma is a kind of benign tumor that originates from smooth muscle cells and is most commonly found in the uterus of women [1]. Leiomyoma can also occur in the smooth muscles of the gastrointestinal tract and lungs, although the incidence rate is relatively low. Apart from this, leiomyoma hardly ever appear in other organs. However, their varying sizes and types of degeneration make leiomyoma difficult to be detected by imaging examinations, which, in turn, leads to misdiagnosis [2]. Currently, there are very few case reports of leiomyoma occurring in the hepatorenal recess.

## Case report

**Figure 1:**
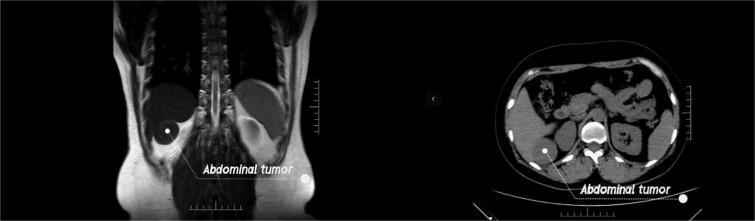
The MRI and the CT examination.

In this video (Video Clip 1), we report a case of a previously misdiagnosed patient with leiomyoma in the hepatorenal recess. A 44-year-old woman found a tumor between the liver and the kidney during a routine physical examination. Although the patient was asymptomatic, the doctor at the physical examination center recommended that patient come to our hospital for further consultation as the nature of the lesion could not be determined. Upon admission, the physical examination revealed no positive fings. The MRI examination identified a tumor in the hepatorenal space, suggestive of a benign tumor. The CT examination indicated an indistinct boundary between the tumor and the liver, suggesting its hepatic origin (Fig. 1). All other laboratory tests showed negative results. Plasma cortisol levels as well as renin and angiotensin were also within normal range. After fully communicating with the patient, it was decided to adopt a surgical treatment plan.

**Figure 2:**
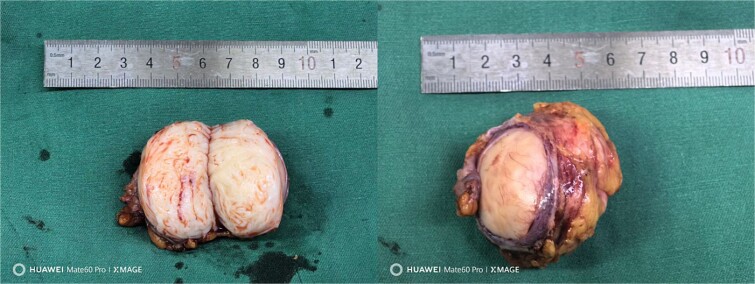
Excision tumor specimen.

**Figure 3:**
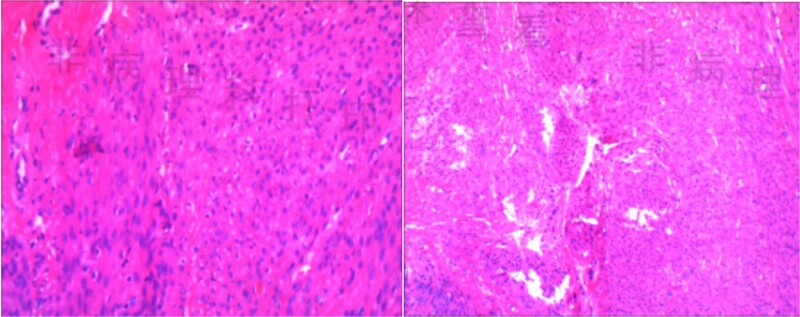
Pathological report.

The patient was placed in the supine position, and five trocars were strategically placed in a U-shaped arrangement around the tumor site prior to resection. Following isolation of the right hepatorenal ligaments, a tough-textured tumor measuring 4.0 × 5.0 cm was identified. After gradually separating the tumor and liver, it was observed that the tumor did not originate from the liver. After further separating the tumor, it was found to be closely related to the right adrenal gland. Therefore, we invited the urology department for a consultation during the operation. Subsequently, we decided to perform a pathological examination of frozen sections after achieving complete resection (Fig. 2). Intraoperative freezing confirmed the presence of a mesenchymal spindle cell tumor, and the specimen was extracted through a surgical incision. The surgical procedure lasted for a duration of 92 min, during which the patient experienced a blood loss of 30 ml. The patient exhibited satisfactory recovery and was discharged on the fourth day post-surgery. The postoperative pathological report confirmed the presence of leiomyoma (Fig. 3). This case report has been published with written informed consent obtained from the patient.

## Discussion

Leiomyoma is a benign myogenic tumor that may develop wherever smooth muscle is present without specific clinical signs or symptoms [3]. It occurs commonly in the uterus, skin, and gastrointestinal tract and is rare within the organ junction. Studies have shown that leiomyomas in deep tissues usually originate from small veins and their behavior is affected by many factors, including age, tumor grade, and “destruction” of the tumor [4]. When the tumor occurs in certain organ spaces, it may compress adjacent organs, resulting in blurred boundaries in imaging examinations and making it difficult to determine its origin. For abdominal tumors lacking typical imaging features, how to make a clear diagnosis and follow-up treatment has become a difficult problem for clinicians.

Due to the rarity and variability of abdominal borderline tumors, preoperative imaging diagnosis is often extremely difficult, and the diagnosis mainly depends on postoperative pathology and immunohistochemistry [5]. Therefore, a perfect evaluation before surgery as well as the design of the surgical approach becomes crucial. As in our case, the surgical plan was communicated with the urologist, and completing the urological examination. Additionally, effective communication with patients is essential.

For undiagnosed abdominal tumors, surgeries must be carried out with great caution to avoid some unnecessary damages [6]. Since we initially thought that the tumor originated from the liver, we adopted a “step-by-step” approach to separate the tissues around the liver. Clear tissue layers are of great help in determining the origin of rare tumors during the operation. As surgery progressed, we applied for a multidisciplinary consultation during surgery when we found that the abdominal tumor did not originate from the liver. Finally, the operation was completed with the assistance of urologists. The postoperative pathological report was leiomyoma.

To our knowledge, this is the first report of leiomyoma in the hepatorenal space. We discussed and analyzed the causes of abdominal tumor which are easily misdiagnosed. There were signs of compression of the liver by the lesion and the boundaries are not clear, so that diagnostician think it most likely originated in the liver. At the same time, the patient’s history, physical examination, and blood chemistry examination did not provide more useful information. These leads to the ambiguity of the diagnosis.

Although the diagnosis is not clear, surgery can still be carried out after thorough communication with the patient, and the diagnosis can be made during the operation. It should be emphasized that multidisciplinary consultations before surgery and meticulous preparation are of vital importance for diseases that are prone to misdiagnosis. When clinicians encounter unknown abdominal tumors, including benign tumors found by imaging, they should consider the possibility of leiomyoma in order to prevent missed diagnoses or misdiagnoses.

## Conclusions

For rare tumors in the junction, it is difficult to diagnose them through imaging examination and other auxiliary examinations in some cases. In order to achieve better therapeutic effect, abdominal exploration and intraoperative multidisciplinary consultation should be performed with sufficient preoperative preparation.

## Supplementary Material

Video_rjaf062

## Data Availability

Not applicable.
